# The tumor suppressor capability of p53 is dependent on non-muscle myosin IIA function in head and neck cancer

**DOI:** 10.18632/oncotarget.14967

**Published:** 2017-02-01

**Authors:** Sonya D. Coaxum, Jessica Tiedeken, Elizabeth Garrett-Mayer, Jeffrey Myers, Steven A. Rosenzweig, David M. Neskey

**Affiliations:** ^1^ Department of Otolaryngology, Head and Neck Surgery, Medical University of South Carolina, Charleston, SC, USA; ^2^ Department of Cell and Molecular Pharmacology & Experimental Therapeutics, Medical University of South Carolina, Charleston, SC, USA; ^3^ Department of Public Health Sciences and Hollings Cancer Center, Medical University of South Carolina, Charleston, SC, USA; ^4^ Department of Head & Neck Surgery, M.D. Anderson Medical Center, Houston, TX, USA

**Keywords:** head and neck squamous cell carcinoma, TP53, non-muscle myosin IIA, tumor suppressor

## Abstract

Over 300,000 patients develop squamous cell carcinoma of the head and neck (HNSCC) worldwide with 25-30% of patients ultimately dying from their disease. Currently, molecular biomarkers are not used in HNSCC but several genes have been identified including mutant *TP53* (mutp53) Our recent work has identified an approach to stratify patients with tumors harboring high or low risk *TP53* mutations. Non-muscle Myosin IIA (NMIIA) was recently identified as a tumor suppressor in HNSCC. We now demonstrate that low *MYH9* expression is associated with decreased survival in patients with head and neck cancer harboring low-risk mutp53 but not high-risk mutp53. Furthermore, inhibition of NMIIA leads to increased invasion in cells harboring wildtype p53 (wtp53), which was not observed in high-risk mutp53 cells. This increased invasiveness of wtp53 following NMIIA inhibition was associated with reduced p53 target gene expression and was absent in cells expressing mutp53. This reduced expression may be due, in part, to a decrease in nuclear localization of wtp53. These findings suggest that the tumor suppressor capability of wtp53 is dependent upon functional NMIIA and that the invasive phenotype of high-risk mutp53 is independent of NMIIA.

## INTRODUCTION

Head and neck squamous cell carcinoma (HNSCC) is the 6^th^ most common cancer worldwide and affects over 60,000 patients annually in the US [1]. Treatment of advanced HNSCC requires complex, multimodality therapy, employing either definitive radiation with or without chemotherapy or surgical resection and post-operative radiation, with chemotherapy for patients with high-risk of recurrence [2, 3]. Currently, there are no molecular biomarkers to guide these management decisions. Multiple studies have demonstrated *TP53* mutations are prognostic for poor outcomes in HNSCC, yet molecular testing for *TP53* alterations has not become routine [4–8]. Our previous work developed and validated a novel method, EAp53, which can stratify patients with tumors harboring *TP53* mutations as low or high risk which is an extension of the Evolutionary Trace (ET) approach, an extensively validated method to identify key functional or structural residues in proteins [9]. In an effort to predict which *TP53* mutations are highly deleterious every sequence position is assigned a grade of functional sensitivity to sequence variations, defined by whether its evolutionary substitutions correlate with larger or smaller phylogenetic divergences. Residues with large ET grades typically cluster structurally into evolutionary ‘hot-spots’ that overlap and predict functional sites [10].

We have demonstrated that the ET method could assess the impact of *TP53* missense mutations. The impact was shown to be greater when the mutated residues were more evolutionarily sensitive to sequence variations, i.e. have a larger ET grade, and also when the amino acid change was least conservative, so the mutational impact is the largest. These two components were computed and combined into a single score, called Evolutionary Action EA [11]. To apply this Evolutionary Action to *TP53* mutations in HNSCC, we further developed a scoring system (EAp53) to stratify *TP53* missense mutations into high and low risk. The subset of oncogenic or high-risk p53 mutations was associated with decreased survival in patients with HNSCC and increased cellular invasion and tumorigenicity [12]. In contrast, low-risk p53 mutations appeared to have retained some p53 function since patients with HNSCC containing these alterations had similar survival outcomes to wildtype p53 and cells had an intermediate level of invasiveness and tumorigenicity [12].

Class 2 myosins include a family of three nonmuscle myosins that are implicated in force generation and cell migration [13, 14]. Class 2 non-muscle myosins are hexameric molecules, comprised of a pair of heavy chains, a pair of essential light chains, and a pair of regulatory light chains (RLCs). The distinction between the three myosin II molecules is their unique heavy chain isoforms but each functions through the binding and contracting of F-actin in an ATP-dependent manner. *MYH9* encodes the heavy chain of nonmuscle myosin IIA protein (NMIIA). Depletion or inactivation of NMIIA consistently leads to an increase in polarized lamellipodia formation and migration (wound healing) with a concomitant decrease in non-polarized, blunt, cylindrical protrusions or lobopodia (cellular protrusions that share functional attributes with lamellipodia and membrane blebs) formation and focal adhesions [15]. This increase in cell migration following suppression or loss of NMIIA function appears to be due to microtubule stabilization and expansion into lamellae, which can be detected by increased acetylation of α-tubulin in epithelial cells [16]. In NMIIA depleted cells, stabilized microtubules within lamellae may be driving migration through activation of Rac1 leading to enhanced actin polymerization at the leading edge [16]. This mechanism of increased migration through NMIIA suppression can be translated clinically as patients with decreased *MYH9* expression have an associated decrease in overall survival [17]. Therefore, further investigation of NMIIA's role in microtubule regulation will be significant by providing the foundation for treatment strategies targeting actively migrating cells.

In addition to NMIIA's role in cell migration, it has also been identified as a tumor suppressor that can modulate wildtype p53 (wtp53) expression. The inhibition or suppression of NMIIA leads to decreased p53 nuclear accumulation and subsequent decreases in expression of downstream target genes [17]. To date, whether the tumor suppressor capability of p53 is dependent on the function of NMIIA remains unknown. Furthermore, the tumor suppressor characteristics of NMIIA in the context of mutated p53 have yet to be studied. The phenotypic similarities between high-risk mutp53 and NMIIA depleted cells suggests their common oncogenic phenotype may be due, in part, to loss of NMIIA's tumor suppressor function. Therefore, the goal of this study was to determine whether loss of NMIIA function in wtp53 harboring cells reduces its tumor suppressor capability, leading to invasive cell behavior similar to that seen in high-risk mutp53.

## RESULTS

### *MYH9* expression correlates with increased survival in patients with HNSCC having functional p53

**Figure 1:**
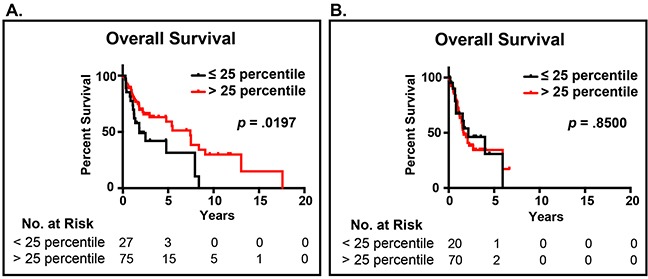
Impact of *MYH9* expression and p53 mutational status **A**. Patients with low-risk (functional) p53 mutations and *MYH9* expression in the lower quartile (<25%) have decreased survival relative to patients with high *MYH9* expression (>25%). **B**. The expression level of *MYH9* did not impact the survival of patients with high-risk (oncogenic) p53 mutations.

Our previous work demonstrated in two cohorts totaling 264 patients, the novel EAp53 classification could identify high-risk p53 mutations associated with decreased survival in patients with head and neck cancer [12]. Furthermore, EAp53 identified low-risk p53 mutations that were similar to wildtype p53 and associated with improved survival outcomes and appear to retain some residual p53 function [12]. EAp53 was applied to the p53 sequence data and subsequently integrated with the *MYH9* RNAseq expression data from The Cancer Genome Atlas Network Head and Neck Project (Table 1) [18]. This analysis revealed patients with low-risk mutp53 and low *MYH9* expression (n=75) had decreased survival outcomes relative to patients with low-risk mutp53 and high *MYH9* expression, *p*=.020 (n=27) (Figure 1A). High (n=70) or low (n=20) *MYH9* expression was not prognostic in patients with high-risk p53 mutations (Figure 1B).

**Table 1:** *TP53* mutations scored and stratified by EAp53 with *MYH9* expression data from The Cancer Genome Atlas HNSCC Project

No.^a^	TCGAID^b^	P53status^c^	Mutation^d^	EA Score^e^	EA Risk^f^	*MYH9*expression^g^	Lower 25^th^percentile^h^
1	7250	Wildtype	NA	0	Low	19059.1436	Yes
2	6441	Wildtype	NA	0	Low	21185.7003	Yes
3	6871	Wildtype	NA	0	Low	22738.9068	Yes
4	4730	Wildtype	NA	0	Low	23025.1362	Yes
5	6939	Wildtype	NA	0	Low	25580.2255	Yes
6	4228	Wildtype	NA	0	Low	26217.3401	Yes
7	6938	Wildtype	NA	0	Low	30394.2155	Yes
8	7406	Wildtype	NA	0	Low	30611.831	Yes
9	7440	Wildtype	NA	0	Low	32681.4044	Yes
10	7631	Wildtype	NA	0	Low	32909.1267	Yes
11	7250	Wildtype	NA	0	Low	34016.2272	Yes
12	7261	Wildtype	NA	0	Low	34079.456	Yes
13	7068	Wildtype	NA	0	Low	36307.9777	Yes
14	7406	Wildtype	NA	0	Low	38976.1036	No
15	6954	Wildtype	NA	0	Low	39143.3037	No
16	6492	Wildtype	NA	0	Low	39459.7833	No
17	5243	Wildtype	NA	0	Low	40087.7311	No
18	6939	Wildtype	NA	0	Low	41136.5435	No
19	7632	Wildtype	NA	0	Low	41442.4973	No
20	7410	Wildtype	NA	0	Low	41670.6444	No
21	6938	Wildtype	NA	0	Low	42522.8748	No
22	5247	Wildtype	NA	0	Low	42815.4169	No
23	5625	Wildtype	NA	0	Low	42879.4466	No
24	7774	Wildtype	NA	0	Low	43144.2177	No
25	6955	Wildtype	NA	0	Low	44674.5453	No
26	5325	Wildtype	NA	0	Low	45864.7399	No
27	5355	Wildtype	NA	0	Low	49493.0351	No
28	5149	Wildtype	NA	0	Low	50893.2238	No
29	6227	Wildtype	NA	0	Low	52429.5174	No
30	7429	Wildtype	NA	0	Low	52733.2198	No
31	7373	Wildtype	NA	0	Low	53396.9971	No
32	7261	Wildtype	NA	0	Low	54162.1353	No
33	6010	Wildtype	NA	0	Low	54341.1575	No
34	7407	Wildtype	NA	0	Low	54573.2791	No
35	7392	Wildtype	NA	0	Low	55223.3527	No
36	7832	Wildtype	NA	0	Low	56462.9173	No
37	7427	Wildtype	NA	0	Low	61582.7367	No
38	7367	Wildtype	NA	0	Low	61906.8592	No
39	7395	Wildtype	NA	0	Low	63655.5958	No
40	5369	Wildtype	NA	0	Low	64139.3305	No
41	55565369	Wildtype	NA	0	Low	65945.6815	No
42	74405556	Wildtype	NA	0	Low	66238.6672	No
43	70857440	Wildtype	NA	0	Low	73182.8255	No
44	71837085	Wildtype	NA	0	Low	77566.3146	No
45	71837183	Wildtype	NA	0	Low	77566.3146	No
46	74117183	Wildtype	NA	0	Low	85911.796	No
47	60037411	Wildtype	NA	0	Low	91717.6666	No
48	73976003	Wildtype	NA	0	Low	94650.3973	No
49	74017397	Wildtype	NA	0	Low	102414.7452	No
50	40747401	Mutant	p.E258D	57.73	Low	14600.1858	Yes
51	6962	Mutant	p.Y236C	62.93	Low	20903.5941	Yes
52	5332	Mutant	p.P151S	64.12	Low	20955.9877	Yes
53	6225	Mutant	p.E224D	39.02	Low	21525.3994	Yes
54	4076	Mutant	p.Q136P	71.29	Low	22118.8508	Yes
55	5329	Mutant	p.R273H	66.12	Low	22833.8745	Yes
56	7245	Mutant	p.R282W	73.21	Low	24809.4695	Yes
57	5973	Mutant	p.R282W	73.21	Low	25385.2785	Yes
58	7424	Mutant	p.E271V	74.39	Low	25942.2319	Yes
59	7437	Mutant	p.S106R	21.82	Low	27579.5418	Yes
60	7423	Mutant	p.A159V	62.4	Low	27604.2023	Yes
61	6436	Mutant	p.R273H	66.12	Low	31219.2933	Yes
62	6962	Mutant	p.Y236C	62.93	Low	35420.671	Yes
63	6951	Mutant	p.Y234C	62.94	Low	35732.5631	Yes
64	7398	Mutant	p.R337L	61.55	Low	36115.016	Yes
65	4736	Mutant	p.I195T	72.13	Low	37253.7068	No
66	4740	Mutant	p.M237I	63.68	Low	37284.3268	No
67	6933	Mutant	p.P151H	71.97	Low	38325.6981	No
68	7238	Mutant	p.R273H	66.12	Low	39422.9096	No
69	7592	Mutant	p.Y220C, p.R110L	72.52, 28.14	Low	39492.7307	No
70	7630	Mutant	p.139_142KTCP>T, p.M1V	43.89	Low	40807.4754	No
71	5430	Mutant	p.R282W, p.P89fs	73.21,	Low	41157.1652	No
72	7424	Mutant	p.E271V	74.39	Low	41401.4767	No
73	7437	Mutant	p.S106R	21.82	Low	43367.2117	No
74	4739	Mutant	p.R337C	63.66	Low	44781.6908	No
75	5151	Mutant	p.V143M	51.72	Low	44811.7412	No
76	4217	Mutant	p.R158L	57.61	Low	44929.7646	No
77	7238	Mutant	p.R273H	66.12	Low	44945.2333	No
78	5978	Mutant	p.V172F	65.55	Low	45299.2832	No
79	7414	Mutant	p.E285K	69.87	Low	47003.5151	No
80	7235	Mutant	p.F270C, p.T211I	66.32, 68.48	Low	47573.0464	No
81	6013	Mutant	p.R282W	73.21	Low	48053.8936	No
82	7374	Mutant	p.R273H	66.12	Low	49671.3671	No
83	7099	Mutant	p.E285K	69.87	Low	49963.8243	No
84	5434	Mutant	p.Y236C, p.R213*	62.93	Low	53941.7304	No
85	4737	Mutant	p.H168L	62.62	Low	54291.7232	No
86	7235	Mutant	p.F270C, p.T211I	66.32, 68.48	Low	54443.1599	No
87	5334	Mutant	p.S166*, p.R158H	43.94	Low	55111.0587	No
88	7089	Mutant	p.Y163C	70	Low	60442.0202	No
89	6933	Mutant	p.P151H	71.97	Low	62108.8343	No
90	7394	Mutant	p.R273H	66.12	Low	64876.995	No
91	5629	Mutant	p.V157F	55.26	Low	66941.442	No
92	7423	Mutant	p.A159V	62.4	Low	67549.236	No
93	5366	Mutant	p.P151T	70.26	Low	71424.9734	No
94	7435	Mutant	p.Y220C	72.52	Low	72632.5124	No
95	7380	Mutant	p.R282W	73.21	Low	77702.8389	No
96	7588	Mutant	p.L137Q	64.66	Low	80069.0459	No
97	4733	Mutant	p.R273H	66.12	Low	80139.2593	No
98	7236	Mutant	p.V143M	51.72	Low	85991.952	No
99	7365	Mutant	p.V216M	73.3	Low	87008.6792	No
100	6221	Mutant	p.V272M	63.49	Low	98089.8266	No
101	7245	Mutant	p.R282W	73.21	Low	112385.1618	No
102	7090	Mutant	p.R273H	66.12	Low	128727.6925	No
103	5370	Mutant	p.R175H, p.Y126_splice	78.51,	High	17991.2352	Yes
104	6023	Mutant	p.G245S	86.45	High	20435.1948	Yes
105	7178	Mutant	p.C176Y, p.R110L	93.11, 28.14	High	22902.4691	Yes
106	5152	Mutant	p.G245S	86.45	High	24189.1074	Yes
107	6943	Mutant	p.R248W	84.11	High	24203.8835	Yes
108	7065	Mutant	p.H179P	98.89	High	24548.4018	Yes
109	6934	Mutant	p.Y205C	77.88	High	24737.1678	Yes
110	7242	Mutant	p.V173M	75.53	High	25359.3433	Yes
111	7254	Mutant	p.E258A	93.29	High	26404.9051	Yes
112	6959	Mutant	p.R248W	84.11	High	26977.7238	Yes
113	7418	Mutant	p.H179Y	77.78	High	29714.3994	Yes
114	7370	Mutant	p.C238S	86.53	High	31748.1805	Yes
115	6869	Mutant	p.C238F, p.R156P	96.54, 42.93	High	31750.7152	Yes
116	7399	Mutant	p.P278S, p.R213L	84.34, 90.71	High	33442.556	Yes
117	7082	Mutant	p.R248W	84.11	High	34283.7838	Yes
118	6936	Mutant	p.V173L	82.64	High	34347.8836	Yes
119	6935	Mutant	p.C242S	86.74	High	34914.732	Yes
120	7848	Mutant	p.E286V, p.P58fs	94.09	High	35968.0861	Yes
121	7413	Mutant	p.G105C	90.8	High	36065.8762	Yes
122	7263	Mutant	p.Y126C	81.09	High	36794.0111	Yes
123	5558	Mutant	p.R282W, p.R175H	73.21, 78.51	High	39755.88	No
124	6992	Mutant	p.Q331H, p.R249M, p.G245D	9.79, 95.41, 89.56	High	40108.2414	No
125	6870	Mutant	p.C242Y	93.46	High	40158.9134	No
126	5444	Mutant	p.R248Q, p.G245S	78.95, 86.45	High	40765.04	No
127	6936	Mutant	p.V173L	82.64	High	41410.0741	No
128	7242	Mutant	p.V173M	75.53	High	41666.9217	No
129	7248	Mutant	p.C242F	97.04	High	43282.1983	No
130	6945	Mutant	p.H193L	95.4	High	43425.0326	No
131	5431	Mutant	p.H193P, p.H179Y	92.46, 77.78	High	43788.9126	No
132	4725	Mutant	p.C275F	97.06	High	44576.836	No
133	6872	Mutant	p.R175H	78.51	High	45312.0393	No
134	6493	Mutant	p.C229fs, p.S127Y	87.62	High	45516.3524	No
135	7371	Mutant	p.R175H	78.51	High	45522.7596	No
136	5373	Mutant	p.G245V	98.74	High	45758.6207	No
137	7402	Mutant	p.R267P	88.48	High	45787.4794	No
138	6824	Mutant	p.K132N	92.16	High	45810.6559	No
139	6478	Mutant	p.H179R	81.91	High	45909.8192	No
140	7368	Mutant	p.R248Q	78.95	High	46323.4255	No
141	6935	Mutant	p.C242S	86.74	High	46893.4093	No
142	5331	Mutant	p.A307_splice, p.R280T	96.08	High	47291.6078	No
143	7416	Mutant	p.R248Q	78.95	High	47571.3318	No
144	7415	Mutant	p.M133K	93.62	High	47578.0949	No
145	4729	Mutant	p.H179R, p.V157F	81.91, 55.26	High	48687.3857	No
146	5966	Mutant	p.V173M	75.53	High	49092.3772	No
147	6218	Mutant	p.V218G, p.L194fs	89.92	High	49304.6974	No
148	7388	Mutant	p.R273C	84.52	High	49483.4242	No
149	7379	Mutant	p.G262V, p.Q136H	88.02, 47.50	High	49736.8282	No
150	6952	Mutant	p.C275F	97.06	High	49779.8418	No
151	5631	Mutant	p.E336*, p.G245S	86.45	High	50265.4975	No
152	6012	Mutant	p.Y126S	94.81	High	50330.1475	No
153	6020	Mutant	p.C176S	86.9	High	51044.6781	No
154	4723	Mutant	p.C242F	97.04	High	52741.6378	No
155	7376	Mutant	p.R280S, p.L32_splice	94.74	High	52830.2812	No
156	5436	Mutant	p.G266E, p.E56*	93.08	High	53609.38	No
157	6024	Mutant	p.L265R	84.18	High	54654.3939	No
158	7416	Mutant	p.R248Q	78.95	High	55950.4367	No
159	7372	Mutant	p.R248W	84.11	High	56103.6943	No
160	7219	Mutant	p.R196P	95.55	High	59325.7308	No
161	6011	Mutant	p.P278S, p.Y205fs	84.34	High	59562.9966	No
162	5365	Mutant	p.H193L	95.4	High	59633.7932	No
163	6491	Mutant	p.M237V, p.H179R	75.79, 81.91	High	61669.9501	No
164	6516	Mutant	p.G262V	88.02	High	61964.9147	No
165	6022	Mutant	p.S261_splice, p.R248W	84.11	High	62102.963	No
166	4738	Mutant	p.Q331*, p.H179Y	77.78	High	62189.1787	No
167	6220	Mutant	p.R280G	95.71	High	62341.3455	No
168	5367	Mutant	p.R273C, p.A161T	84.52, 58.51	High	62735.4238	No
169	7178	Mutant	p.C176Y, p.R110L	93.11, 28.14	High	64539.6273	No
170	7229	Mutant	p.R249S	93.65	High	65436.9925	No
171	5976	Mutant	p.Y236D	92.17	High	70703.2873	No
172	6018	Mutant	p.R248W	84.11	High	70937.0085	No
173	5970	Mutant	p.R248Q	78.95	High	75136.8374	No
174	5330	Mutant	p.G266R	91.41	High	77750.4254	No
175	6517	Mutant	p.S127F	88.07	High	83366.1651	No
176	6943	Mutant	p.R248W	84.11	High	83366.6878	No
177	7102	Mutant	p.G266E	93.08	High	84155.9398	No
178	7421	Mutant	p.R175H	78.51	High	84446.3616	No
179	5979	Mutant	p.R248Q	78.95	High	84769.3762	No
180	6994	Mutant	p.R283P, p.R175H	75.75, 78.51	High	85123.8984	No
181	6934	Mutant	p.Y205C	77.88	High	85532.0304	No
182	6224	Mutant	p.R175H	78.51	High	86128.7828	No
183	6959	Mutant	p.R248W	84.11	High	86287.8985	No
184	6873	Mutant	p.H193L, p.PHHERC177del	95.4	High	87361.6071	No
185	6016	Mutant	p.G245S	86.45	High	89802.686	No
186	6826	Mutant	p.V173G	93.47	High	95987.892	No
187	6868	Mutant	p.L194P	79.72	High	98740.1575	No
188	5555	Mutant	p.H193R	85.96	High	103716.6397	No
189	7389	Mutant	p.P278S	84.34	High	104961.0553	No
190	5326	Mutant	p.R249S, p.L32_splice	93.65	High	107124.1051	No
191	7753	Mutant	p.E286K	76.21	High	118776.7221	No
192	6474	Mutant	p.G245V	98.74	High	152088.3031	No

a. The number of patients included in the analysis

b. The short ID extracted from The Cancer Genome Atlas Head and Neck Project

c. P53 status delineated as either wildtype or mutant

d. Denotes the specific mutation for each patient, wildtype is delineated as NA

e. Evolutionary Action score from 0-100 with higher scores representing more deleterious mutations. Wildtype p53 (wtp53) sequences were scored as zero since this is the normally functioning protein.

f. Evolutionary Action Risk was determined as previously described but a score greater than 77.78 was consider high-risk [12].

g. Level of *MYH9* expression extracted from The Cancer Genome Atlas Head and Neck Project RNA seq data

h. Low *MYH9* expression was defined as the lower 25^th^ percentile while high *MYH9* expression was defined as greater the 25^th^ percentile.

### P53 function is dependent upon a functional NMIIA

**Figure 2:**
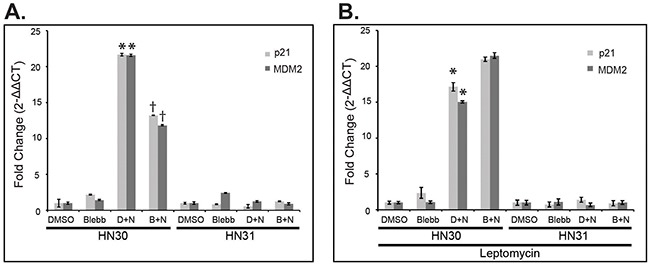
NMIIA is necessary for wtp53 function, which is lost in high-risk mutp53 **A**. After treatment with DMSO, blebbistatin, or a combination of DMSO + nutlin (D+N) or blebbistatin + nutlin (B+N), qRT-PCR revealed the induction of p53 target genes *p21* (*p* = 0.02) and *MDM2* (*p* = 0.04) were significantly reduced following blebbistatin treatment in HN30 cells but not in HN31 cells. **B**. Inhibition of Crm1 nuclear exporter with Leptomycin B rescued p53 target gene expression in HN30 cells. Data expressed as means ± standard deviation; n=3. * *p*<0.05 reduction in *p21* and *MDM2* expression following blebbistatin treatment.

Using the isogenic HNSCC cell lines, HN30 and HN31, which endogenously express either wtp53 (HN30) or missense p53 mutations, C176F and A161S, (HN31), HN30 was shown to upregulate expression of downstream p53 targets *CDKN1A* (*p21*) and *MDM2* following treatment with nutlin-3; which inhibits the interaction between mdm2 and wild type p53, therefore stabilizing and leading to increased levels of the p53 protein. This target gene upregulation is not observed with the mutp53 cell line, HN31 (Figure 2A). NMIIA has been shown to be essential for nuclear retention of activated p53 therefore to determine the impact of NMIIA function on the upregulation of target gene expression observed in the wtp53 cells, the selective, small molecule NMIIA ATPase inhibitor, blebbistatin was applied prior to activation of p53 with nutlin-3. NMIIA inhibition led to a significant reduction in expression of target genes *p21* (*p*=.02) and *MDM2* (*p*=.04) in wtp53, HN30 cells, which was not observed in HN31 cells harboring high-risk mutations (Figure 2A). Inhibiting the nuclear export transporter Crm1 restored target gene expression in wtp53 expressing cells, which was not observed in high-risk mutp53 (Figure 2B). Taken together this data implies with NMIIA is defective, wtp53 cannot activate target genes because of an inability to accumulate within the nucleus.

**Figure 3:**
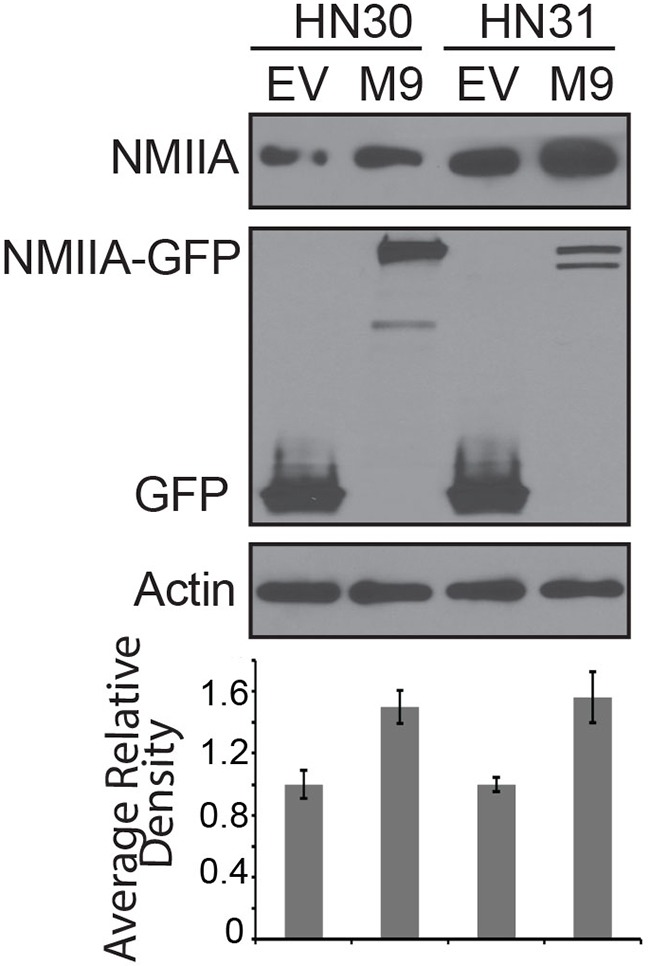
Western blot of cell lines stably expressing EGFP-NMIIA construct The histogram represents average relative density of NMIIA protein expression compared to actin loading controls and is the results of three independent experiments. EV:empty vector; M9:EGFP-NMIIA vector.

**Figure 4:**
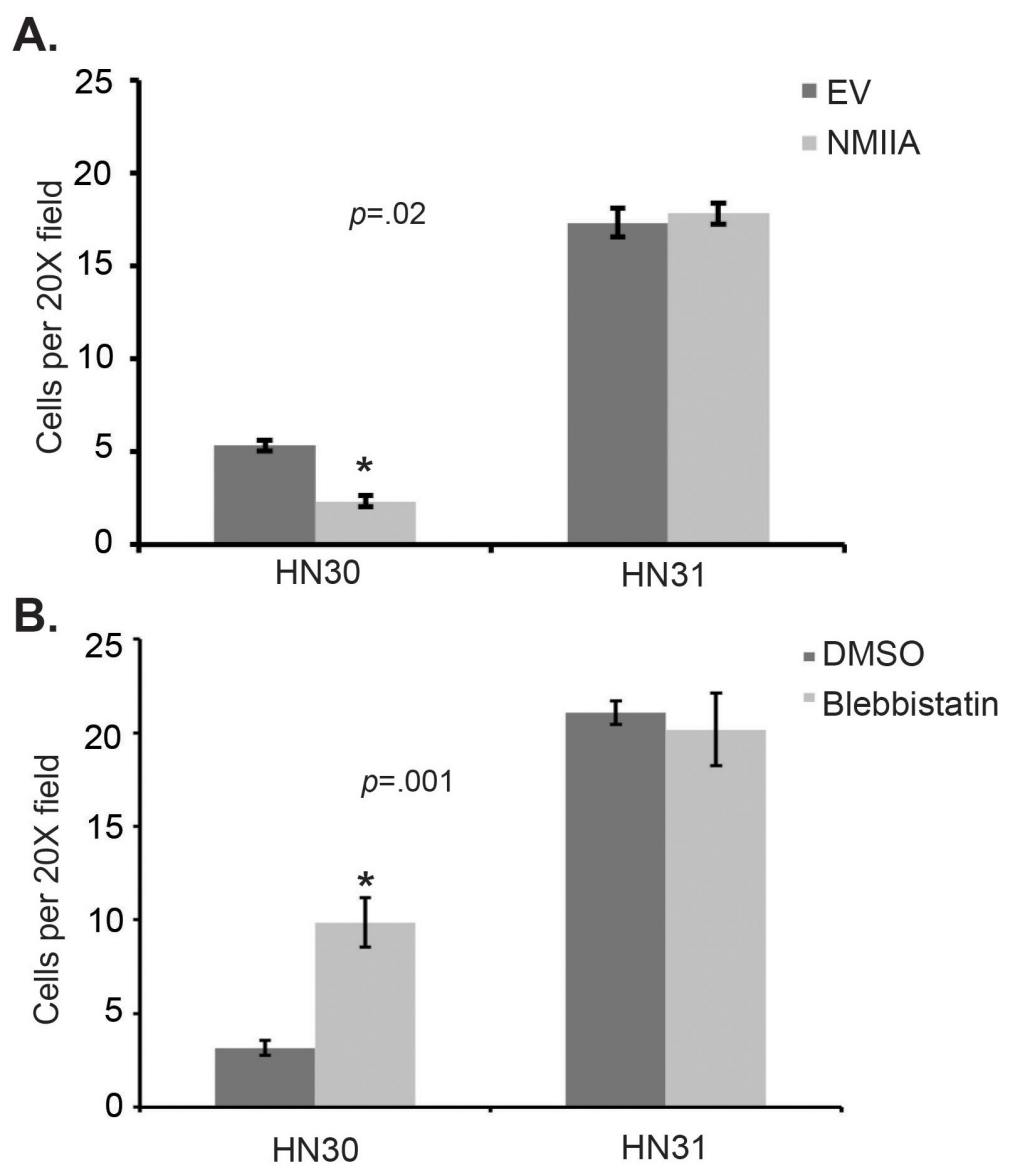
Modulation of NMIIA expression or function alters wtp53 expressing cell invasion **A**. Forced NMIIA expression significantly reduced invasion in HN30 (wtp53) but not HN31 (high-risk mutp53) cells relative to vector controls, *p*=0.02.EV: empty vector control. **B**. NMIIA inhibition significantly increased invasion in HN30 but not HN31 cells, *p*=0.001.

In an effort to directly assess the impact of NMIIA function on cell invasion, a CMV-GFP-NMII-A plasmid was stably overexpressed in HN30 and HN31 cell lines resulting in a ~50% increase in NMIIA expression in both cell lines (Figure 3) [19]. Even this modest (<2 fold) NMIIA overexpression preferentially decreased invasion in cells harboring wtp53 (*p*=.02) which was not observed in the mutp53 cells (Figure 4A). In contrast, inhibition of NMIIA led to an increase in cellular invasion in wtp53 expressing HN30 cells (*p=*.001) but not high-risk mutp53 HN31 cells (Figure 4B). Taken together this data suggests the function of wildtype p53 as a transcription factor and regulating cell invasion is dependent on a functional NMIIA.

### Inhibition of NMIIA alters wtp53 but not mutp53 function and cellular localization

**Figure 5:**
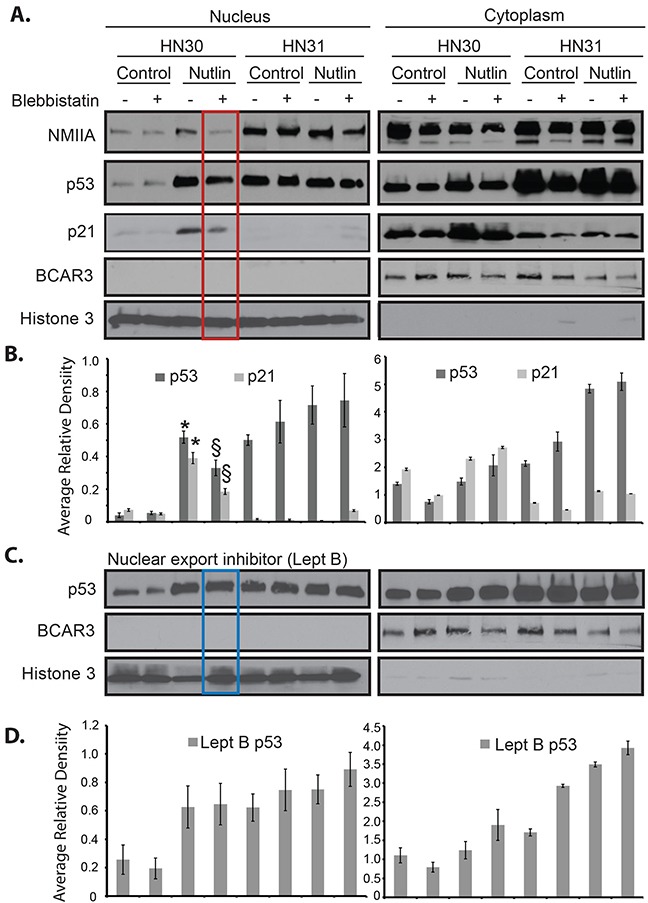
Inhibition of NMIIA alters wtp53 but not high-risk mutp53 cellular localization **A**. Nutlin-induced nuclear / cytoskeletal p53 and p21 was detected in HN30 (wtp53) cells. Blebbistatin treatment attenuated the effect of nutlin on nuclear p53 and p21 induction (red box). **B**. Average relative density in the nuclear / cytoskeletal and cytosolic fractions normalized the level of p53 and p21 to Lamin B and BCAR3 respectively. This revealed a significant increase in p53 and p21 after nutlin treatment relative to control (* *p*=.010), along with a significant decrease in p53 and p21 after blebbistatin relative to control (§ *p*=.032). **C**. Nuclear accumulation of p53 was restored by leptomycin B (Lept B) treatment. **D**. Average relative density in the nuclear / cytoskeletal and cytosolic fractions normalized the level of p53 to Lamin B and BCAR3 respectively. The expression levels of p53 in HN31 (mutp53) cells was unaffected by nutlin, blebbistatin, or leptomycin B treatment. The histograms represent the cumulative results of three independent experiments.

**Figure 6:**
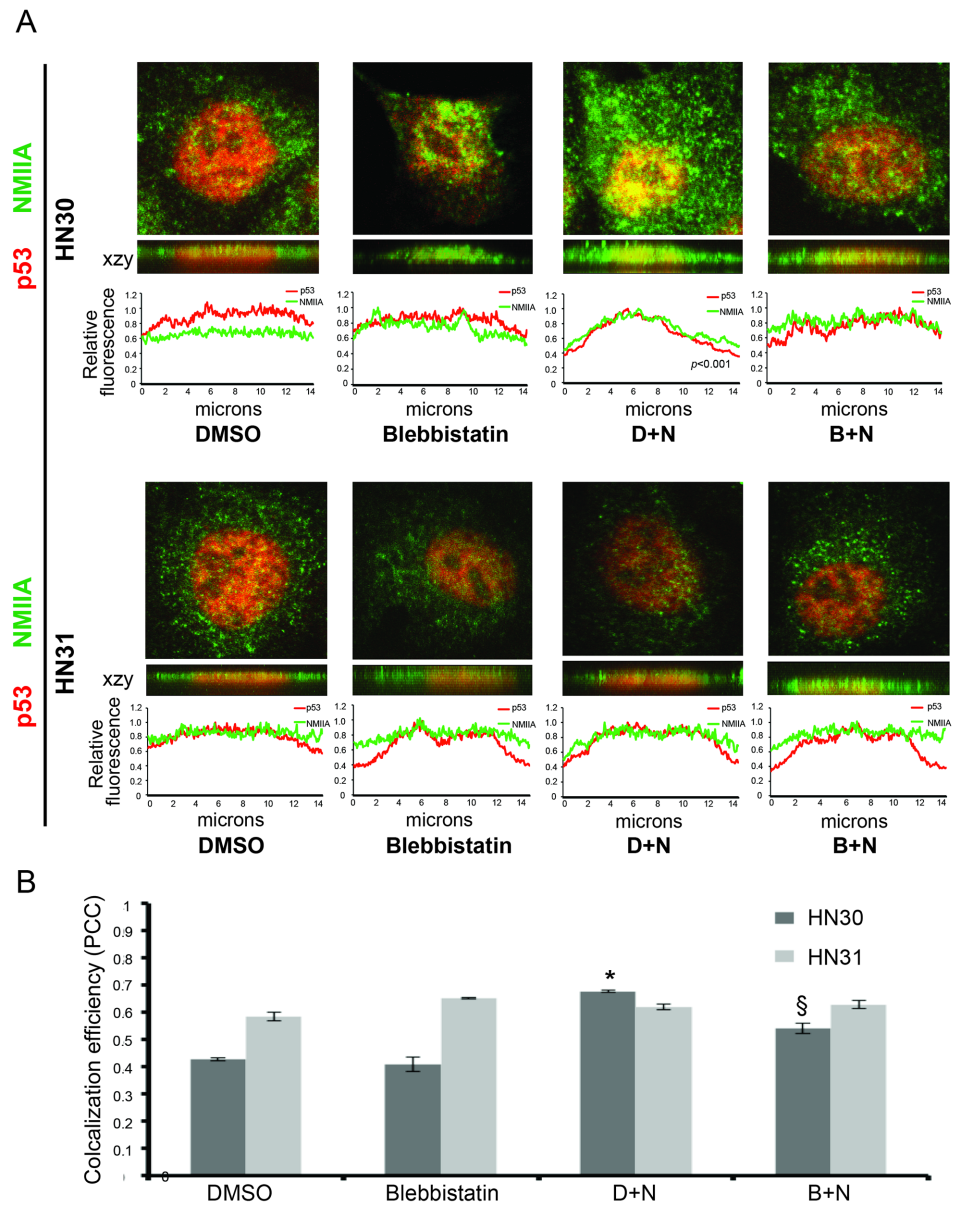
NMIIA co-localization with wtp53 is attenuated following NMIIA inhibition **A**. Representative confocal fluorescence images including Z stack generated orthogonal views (xzy) showed the colocalization of wtp53 and NMIIA in HN30 cells not seen in high-risk HN31 cells (mutp53). The relative immunofluorescence profile revealed a significant increase in nuclear colocalization of p53 / NMIIA following nutlin treatment (*p*<0.001) which is attenuated following NMIIA inhibition. **B**. Data summary shows colocalization efficiency of NMIIA and p53. Nutlin treatment (D+N) caused a significant increase in colocalization in HN30 (p<0.001) but not HN31 (p=0.179) cells. There was a significant reduction in colocalization in HN30 cells following blebbistatin treatment (B+N) not observed in HN31 cells (p=0.019 vs .25). Data expressed as means +/− SEM; n=3. * p<.05 versus DMSO control group; § p<0.05 (DMSO + nutlin versus blebbistatin + nutlin). D+N, DMSO +nutlin; B+N, blebbistatin + nutlin.

Differences in NMIIA's effect on wtp53 *vs*. mutp53 remain unknown [17]. To determine if the selective effect of NMIIA on wtp53 is due to its role in nuclear retention of activated wtp53 but not mutp53, cell fractionation was utilized. The initial fractionation experiment isolated insoluble cellular components (nuclear and cytoskeletal) from soluble cellular components (cytosol). As shown in Figure 5A (red boxed lane) following a dual nutlin-3 / blebbistatin treatment a decrease in nuclear / cytoskeletal expression of wtp53 and reduced induction of p21 was observed. The same treatment in mutp53 cells had no effect on the nuclear / cytoskeletal fraction of p53 or target gene induction (Figure 5). To assess if NMIIA specifically effects the nuclear retention of wtp53, the nuclear export receptor Crm1 was inhibited which resulted in the restoration of p53 nuclear accumulation (Figure 5B, boxed blue lane). To validate these findings a second fractionation protocol was utilized that specifically extracts the nuclear fraction from the cytoskeletal and cytoplasmic fractions. As seen in Supplementary Figure 1, inhibition of NMIIA following nutlin treatment significantly reduced the nuclear accumulation of wtp53 and p21 induction (Supplementary Figure 1). Furthermore, this decrease in nuclear p21 induction following combined nutlin-3 / blebbistatin treatement inhibition was associated with an significant increase in cytosolic p21 induction (Supplementary Figure 1). To confirm these findings immunofluorescent staining of intact cells following nutlin-3 treatment was performed. As shown in Figure 6, we observed a significant increase in co-localization of wtp53 and NMIIA in HN30 cells following nutlin treatment (*p*<.001) as determined by Pearson's correlation coefficient (Figure 6B) and depicted by the yellow staining in the representative confocal images of nutlin treated HN30 cells (Figure 6A). Furthermore, in blebbistatin treated cells co-localization of wtp53 and NMIIA was attenuated. To determine if the wtp53 / NMIIA co-localization was occurring within the nucleus, the relative fluorescence for individual cells was determined and the average fluorescence for p53 and NMIIA was quantified in the cytoplasm and nucleus (2 and 7 microns from the cell membrane edge respectively (Figure 6A). Additionally orthogonal images were constructed from Z stack image capture through the depth of each cell. These analyses revealed that following nutlin-3 treatment, wtp53 and NMIIA appear to co-localize within the nucleus, which is attenuated following blebbistatin treatment supporting the finding that nuclear retention of wtp53 requires a functional NMIIA (Figure 6A and 6B column B+N). While co-localizatiohn of NMIIA and mutp53 was also observed it appeared to be independent of p53 and NMIIA activity given that treatment with either nutlin-3 or blebbistatin did not alter their co-localization.

**Figure 7:**
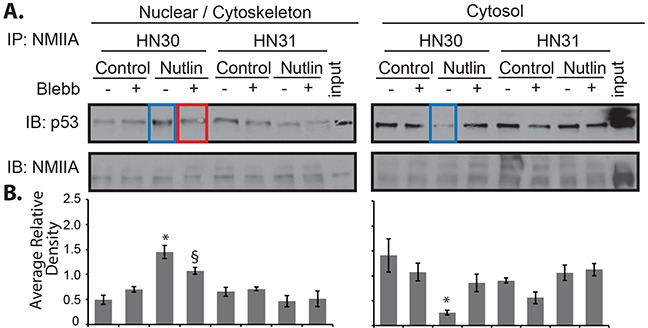
NMIIA exhibits increased interaction with wtp53 in the nucleus HN30 and HN31 cells were treated with blebbistatin (Blebb) or control (PBS) followed by nutlin for 8 h. Cells were fractionated followed by co-immunoprecipitation:immunoblot analysis of NMIIA and p53. **A**. Following p53 activation with nutlin there was a significant increase in association between wtp53 / NMIIA in the nuclear / cytoskeletal fraction of HN30 cells and a concomitant decrease in association in the cytosolic fraction, p<.001 and 0.01, respectively (Blue highlight). The increased association in the nuclear / cytoskeletal fraction was significantly reduced by blebbistatin, p=0.02 (Red highlight). Neither nutlin or blebbistatin treatment had an effect on the nuclear / cytoskeletal or cytosolic mutp53/NMIIA interaction. **B**. Average relative density normalizes the level p53 to NMIIA. The histograms represent the results of three independent experiments. * Significant change in interaction after nutlin treatment relative to control. § Significant decrease in p53/NMIIA interaction after blebbistatin relative to nutlin.

To confirm the nuclear co-localization of wtp53 and NMIIA, cell fractionation followed by direct co-immunoprecipitation from these fractions was performed. This approach revealed an increase in wtp53/NMIIA association in the nuclear / cytoskeletal fraction along with a concomitant decrease in cytosolic interaction following nutlin treatment (Figure 7 blue box lanes). The nuclear / cytoskeletal association of wtp53 / NMIIA was reduced in cells treated with blebbistatin (Figure 7 red boxed lane). As observed by immunofluorescence microscopy, there appears to be an association of mutp53 / NMIIA based on co-immunoprecipitation, but this interaction remained at basal levels following addition of nutlin and/or combined treatment with blebbistatin.

## DISCUSSION

*TP53* is the most frequently mutated gene in HNSCC occurring in more than 70% of cases that are non-human papilloma virus related [18, 20, 21]. Whereas most alterations involving tumor suppressor genes render them nonfunctional through truncation or deletions, p53 is unique in that there is a strong selection bias for missense mutations, particularly within its DNA-binding domain. P53 mutation can result in loss of wild type functions (LOF), which are considered low-risk, through loss of DNA-binding activity to p53 responsive elements or a dominant negative effect where the mutated allele binds and inhibits the remaining functional wild-type allele [22]. Moreover, some mutp53 display oncogenic properties, termed “gain of function” (GOF) or high-risk mutations, which are independent of the loss of wild-type p53 function [23]. Accordingly, GOF p53 mutants can enhance cell transformation, increase tumor formation in mice and confer cellular resistance to chemotherapy [24, 25]. We previously developed and validated a novel method, EAp53 that stratifies patients with tumors harboring *TP53* mutations as high or low risk. Although the underlying mechanisms responsible for high-risk mutp53 remain unresolved, a potential mechanism involves interaction with NMIIA. In addition to the critical role NMIIA has in cell contractility and migration, it also functions as a tumor suppressor through regulation of p53 stability and nuclear retention [15–17, 26]. Despite this novel finding, there continues to be a significant gap in the understanding of the impact of NMIIA on mutp53 and its ability to function as a tumor suppressor and/or contribute to the oncogenic phenotype of p53. Given this lack of understanding, the objective of this study was to correlate the tumor suppressor effects of p53 with NMIIA function and demonstrate NMIIA dysfunction in cells harboring wildtype p53 results in characteristics resembling high-risk mutp53 including increased invasion. We hypothesized that the tumor suppressor capability of p53 is dependent on NMIIA function, which when abrogated leads to an oncogenic phenotype of p53 that is similar to high-risk mutp53.

Our results show patients stratified by EAp53 with low-risk mutp53 had a decreased overall survival with low *MYH9* expression relative to those patients with low-risk mutp53 and high *MYH9* expression. In contrast, the relative expression of *MYH9* did not impact survival in patients with high-risk mutp53. Our previous work demonstrated low-risk *TP53* mutations appear to retain some residual wildtype *TP53* function as demonstrated by an intermediate level of activation of downstream p53 target genes following treatment with cisplatin [27]. Furthermore, this intermediate activation was associated with decreased cell migration and tumor growth in animal models [12]. Taken together, these data indicate that the tumor suppressive capability of NMIIA appears to be confined to tumor cells with functional *TP53*.

In addition to identifying the potential prognostic significance of *MYH9* expression in low-risk mutp53 disease, we demonstrated inhibition of NMIIA leads to increased invasion in wtp53 expressing cells but not in high-risk mutp53 expressing cells. Furthermore, overexpression of NMIIA reduced invasion only in cells expressing wildtype p53. These findings corroborate a previous study and support our hypothesis that the tumor suppressor capability of p53 is dependent on NMIIA function [17]. This hypothesis is further supported by the finding of reduced p53 target gene expression in wildtype p53 cells following NMIIA inhibition, which was not observed in high-risk mutp53. The ability of wildtype p53 to activate downstream target genes appears to be dependent on nuclear localization of p53 as the reduction of target gene expression following NMIIA inhibition could be reversed with nuclear export inhibition. Furthermore, cell fractionation studies revealed induction of p53 and p21 in the nuclear fraction by nutlin treatment of wtp53 cells can be attenuated with blebbistatin treatment. The decrease in nuclear p21 induction was associated with a concomitant increase in the cytosolic p21 level which has been associated with increased cell survival and proliferation [28] This reduction in p53 induction in wtp53 cells with inhibition of the NMIIA ATPase can be reversed with Crm1 inhibition, which supports published data [17]. In contrast, inhibition of NMIIA did not alter expression of p21 or MDM2 in mutp53 cells or retention of mutp53 within the nucleus.

These findings are supported by immunofluorescence microscopy demonstrating an increase in the co-localization of wtp53 and NMIIA following nutlin treatment, which was subsequently reduced by NMIIA inhibition. Colocalization was predominantly nuclear as demonstrated by the Z stack generated orthogonal views and the relative cellular immunofluorescence, which was confirmed by cell fractionation:immmunoprecipitation findings. Although mutp53 and NMIIA appeared to co-localize, this was independent of NMIIA ATPase activity and was observed diffusely throughout the cell based on microscopy and supported by cell fractionation data.

In conclusion, the current findings indicate that cells expressing wtp53 are dependent on NMIIA inhibition to become pro-invasive secondary to decreased nuclear accumulation of wtp53 and subsequent reduction in target gene expression. In contrast, cells harboring high-risk mutp53 attain an invasive phenotype independent of NMIIA.

## MATERIALS AND METHODS

### Patient data

Patient dataset from The Cancer Genome Atlas HNSCC Project that had human papilloma virus (HPV)-negative tumors (n=192) were identified and EAp53 was applied to the p53 sequence data [12, 18]. *MYH9* RNAseq expression data from The Cancer Genome Atlas Network Head and Neck Project was subsequently integrated with the p53 sequence data. *MYH9* expression less than or equal to the lower quartile (≤25 percentile) for the entire cohort was considered to be low expression while expression greater than the lower quartile (>25 quartile) was considered to be high expression (Table 1). Overall survival data was extracted from TCGA HNSCC Supplementary Data. Curves describing overall survival were generated by the Kaplan-Meier method. The statistical significance of differences between the actuarial curves were assessed by the log rank test. Overall survival was measured from the date of diagnosis of recurrent disease to the date of death or last contact. Statistical analyses were performed using GraphPad Prism 7.0e (GraphPad Software, Inc., La Jolla, CA) statistical software.

### Cell culture

The isogenic HNSCC cell lines HN30 and HN31 (provided by Dr. John Ensley; Wayne State University) were chosen as they were derived from a pharyngeal primary tumor and lymph node from the same patient. HN30 harbours a wtp53 while HN31 harbours two p53 mutations, C176F (high-risk) and A161S (low-risk). HN30 and HN31 cells were grown in DMEM with high glucose containing 10% FBS, 0.5% penicillin and streptomycin, 2 mM L-glutamine, 1 mM Sodium pyruvate, 85 mg/mL NaCl, 1 mg/mL D-calcium pantothenate, 1 mg/mL choline chloride, 1 mg/mL folic acid, 2 mg/mL i-inositol, 1 mg/mL niacinamide, 1 mg/mL pyridoxine- HCl, 0.1 mg/mL riboflavin, 1 mg/mL thiamine - HCl and non essential amino acids including 0.1 mM glycine, 0.1 mM alanine, 0.1 mM asparagine, 0.1 mM aspartic acid, 0.1 mM glutamic acid, 0.1 mM proline, 0.1 mM serine. The cells were maintained in a 37°C incubator with 95% air and 5% CO_2_

### Drug incubation

HN30 and HN31 cells were growth arrested in serum-free medium for 24 hrs prior to drug treatment. Cells were pretreated with DMSO or 25 μM blebbistatin (Cayman Chemicals 674289-55-5) for 30 min prior to 8 hrs of treatment with 5 μM of nutlin-3 (Sigma-Aldrich N6287). For some Western blot analyses, (where indicated), 20 nM of leptomycin (Cayman Chemical 87081-35-4) was added 30 min prior to pretreatment of cells with DMSO or blebbistatin.

### qRT-PCR

RNA was prepared from HN30 and HN31 cells using High Pure RNA Isolation Kit (Roche Diagnostics). cDNA was synthesized from RNA using iScript cDNA Syntheis Kit (Bio-Rad). The amplified cDNA was used in quantitative real-time PCR using SYBR Green PCR Master Mix (Applied Biosytems). The primer pairs used for analyzing *p21*, *MDM2*, and *GAPDH* were previously published [27]. The primer pairs used were as followed: p21 forward 5′-CGCTAATGGCGGGCTG-3′, reverse 5′-CGGTGACAAAGTCGAAGTTCC-3′; MDM2 forward 5′-ACCTCACAGATTCCAGCTTCG-3′, reverse 5′-TTTCATAGTATAAGTGTCTTTTT-3′; GAPDH forward 5′-TGATGGTACATGACAAGGTGC-3′, GA PDH reverse 5′-ACAGTCCATGCCATCACTGC-3′.

### Generation of stable cell lines

For stable transfections, HN30 and HN31 cells were cultured in 6-well plates until they reached 70-80% confluency. The cells were transfected with CMV-GFP-NMHC II-A (Addgene plasmid # 11347) using 6 μg of using NanoJuice Transfection Reagent in serum-free medium (Novagen) according to the manufacturer's protocol [19]. HN30 and HN31 cells were cultured for 7-14 days in 400 μg/ml of geneticin before being sorted for selection of stable clones.

### Invasion assay

After stably transfecting HN30 and HN31 cells with vectors, invasion studies were conducted using Corning BioCoat Matrigel Invasion Chambers as described by the manufacturer (Corning). Cells were seeded in Matrigel Basement Membrane Matrix inserts in 24-well plates at a density of 2.5×10^4^ cells per well. After 22 hr in a 37°C incubator, cells were fixed with 3% formalin and stained with silver stain. Membranes were washed and allowed to dry before an image was obtained and the number of invaded cells quantified. For studies that involved drug incubation, HN30 and HN31 cells were plated at a density of 2.5×10^4^ cells in medium containing either DMSO or 25 μM of Blebbistatin.

### Cell fractionation and western blot analysis

For Western blot analysis, HN30 and HN31 cells were grown in 100 mm tissue culture dishes. After treatment with the various drugs described, cells were rinsed and then lysed in cytosolic fractionation buffer (5 mM of EDTA, 1 mM of dithiothreitol, 1% Triton X-100 in PBS) supplemented with protease inhibitors (1 mM phenylmethylsulfonyl fluoride, 1 mM NaF, 1 mM Na_3_VO_3_, 1 μg/ml leupeptin, 1 μg/ml aprotinin, and 1 μg/ml pepstatin). After brief centrifugation the supernatants were collected as the cytosolic extract and the pellets were washed and resuspended in nuclear extraction buffer (20 mM of TRIS-HCl, 1% SDS, 5 mM EGTA, 0.5% Triton X-100, 150 mM of NaCl) supplemented with protease inhibitors.

An alternative fractionation protocol was used where after treatment with the various drugs as described, nuclear and cytoplasmic extracts were prepared using the NE-PER Nuclear and Cytoplasmic Extraction Reagents Kit from ThermoFisher Scientific (Catalog #: 78833). Equal amounts of protein sample were loaded per lane on Mini-PROTEAN TGX Precast Gels (Bio-RAD) which were transferred to nitrocellulose membranes following electrophoresis. Blots were incubated with primary antibodies to p53 (Santa Cruz Biotechnology sc-126), p21 (EMD Millipore OP64), NMIIA (Santa Cruz Biotechnology sc-98978), BCAR3 (Bethly Laboratories A301-671A), Lamin B (Santa Cruz sc-6216), GFP (Cell Signaling 2956S), actin (Millipore MAB1501) and subsequently reacted with the corresponding secondary antibodies. All secondary antibodies were horseradish peroxidase conjugates. Blots were developed by Enhanced Chemiluminescence Kit (Thermo Scientific) before exposure to X-ray film. Densitometry was performed using FIJI/Image J software and paired t-tests compared the relative intensities using Microsoft Excel (Microsoft Corp, Redmond, WA) [29].

### Immunoprecipitation

For NMIIA immunoprecipitation, HN30 and HN31 cells grown in 100 mm tissue culture dishes were fractionated as described above equal amounts of nuclear and cytoplasmic proteins were pre-cleared by incubation with protein A/G Sepharose beads for 30 min at 4°C. After brief centrifugation, supernatants were removed and incubated with anti-Myosin 9 antibody (Santa Cruz Biotechnologies sc-98978) overnight. Immunoprecipitates were captured with 60 μl of protein A/G beads at 4°C for 3 hr. Samples were centrifuged and washed three-fold with PBS and proteins were eluted from the beads using 2x Laemmli buffer, boiled for 5 min, and resolved by SDS-PAGE and subsequent immunoblot analysis with mouse monoclonal antibodies for p53 (Santa Cruz Biotechnologies sc-126) and Myosin 9 (Millipore MABT164). Densitometry and statistical analysis were performed as described above.

### Immunofluorescence microscopy

Following the drug treatments described above, cells were fixed with 4% paraformaldehyde for 10 min at room temperature, permeabilized with 0.5% Triton X-100 (Sigma) in PBS for 5 min and non-specific binding sites were blocked with 1% BSA in PBS for 1h. Primary and secondary antibodies were diluted in blocking solution as directed by the manufacturer. Antibodies employed were anti-p53 (1:500; Santa Cruz Biotechnology), anti-NMIIA (1:500; Santa Cruz Biotechnology), anti-rabbit Alexa Flour 488 (Life Technologies) anti-mouse Alexa Fluor 546 (Molecular Probes). Confocal microscopy was performed using an Olympus FV10i laser scanning confocal microscope (Olympus, Tokyo, Japan). Colocalization of NMIIA and p53 was analyzed by FIJI/Image J software with the coloq2 plugin [29]. The plot profile for 8 cells per condition was determined and the mean was relative immunofluorescence was calculated. The average cell diameter was estimated to be 14 microns, there the relative immunofluorescence within the cytoplasm and nucleus was measured 2 and 7 microns from the cell membrane respectively. The average relative immunofluorescence following various treatments within the nucleus and cytoplasm was compared using a paired T-test. The summarized colocalization efficiency data were expressed as Pearson correlation coefficients as previously described [30].

## SUPPLEMENTARY MATERIALS FIGURES AND TABLES


