# Supplementary material for: The tumor suppressor capability of p53 is dependent on non-muscle myosin IIA function in head and neck cancer
Source: Oncotarget. 2017 Feb 1;8(14):22991–3007. doi: 10.18632/oncotarget.14967 (PMC5410280; doi:10.18632/oncotarget.14967)
Supplement: Supplementary file 1 [file oncotarget-08-22991-s001.pdf]

The tumor suppressor capability of p53 is dependent on non-muscle myosin IIA function in head and neck cancer

SUPPLEMENTARY FIGURE

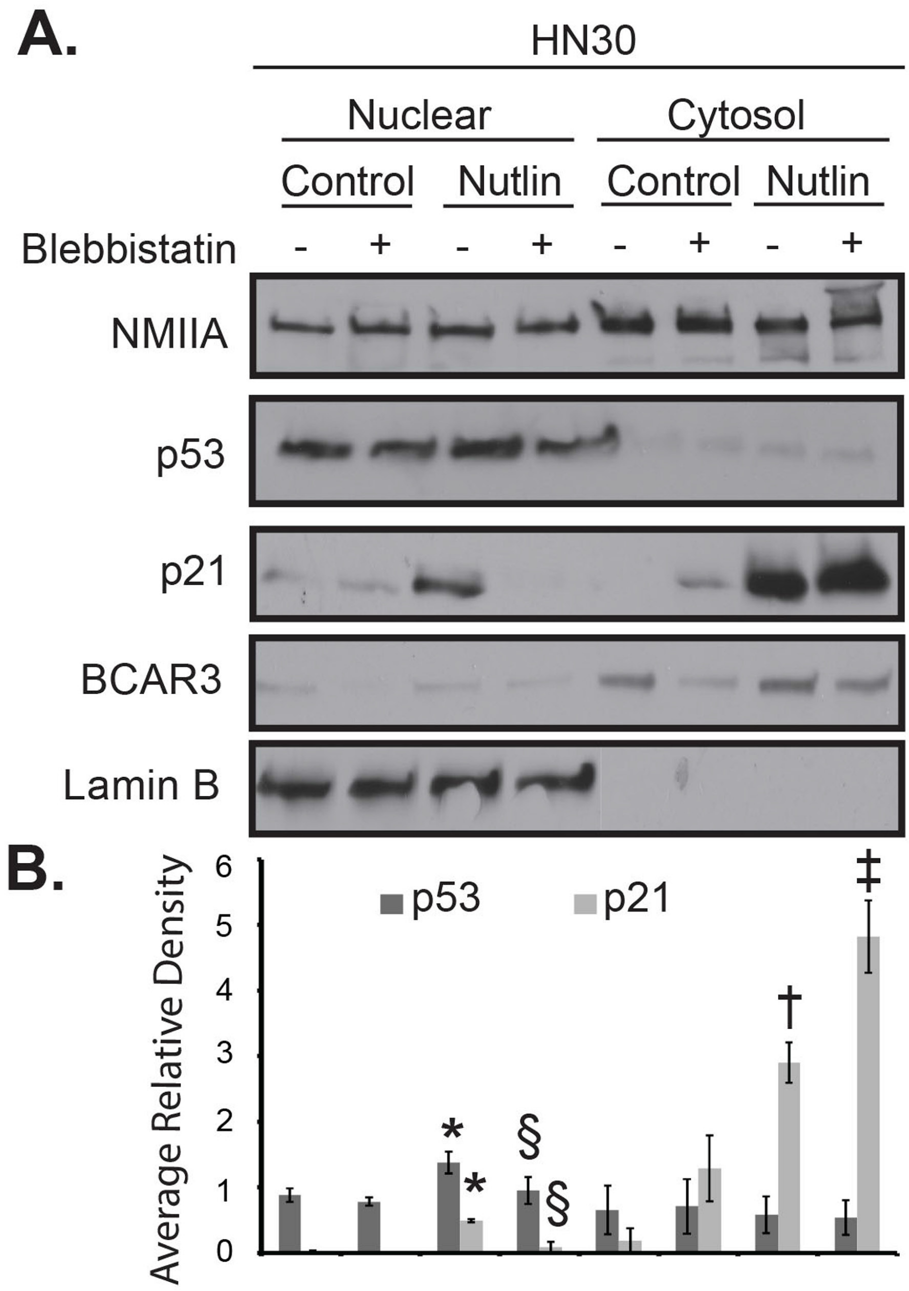

Supplementary Figure 1: NMIIA is required for wtp53 nuclear accumulation. A. Nutlin-induced nuclear p53 and p21 was detected in HN30 (wtp53) cells. Blebbistatin treatment attenuated the effect of nutlin on nuclear p53 and p21 induction. **B.** Average relative density in the nuclear and cytosolic fractions normalized the level of p53 and p21 to Lamin B and BCAR3 respectively. This revealed a significant increase in p53 and p21 after nutlin treatment relative to DMSO (* p=.006), along with a significant decrease in p53 and p21 after blebbistatin relative to nutlin treatment along (§ p=.050). Additionally there was a concomitant increase in the cytosolic p21 level following nutlin treatment alone († p<0.001) and in combination with blebbistatin (‡ p<0.011).
